# IGCLAPS: an interpretable graph contrastive learning method with adaptive positive sampling for scRNA-seq data analysis

**DOI:** 10.1093/bioinformatics/btaf411

**Published:** 2025-07-21

**Authors:** Weihua Zheng, Wenwen Min, Shunfang Wang

**Affiliations:** Department of Computer Science and Engineering, School of Information Science and Engineering, Yunnan University, Kunming 650500, China; Department of Computer Science and Engineering, School of Information Science and Engineering, Yunnan University, Kunming 650500, China; Department of Computer Science and Engineering, School of Information Science and Engineering, Yunnan University, Kunming 650500, China

## Abstract

**Motivation:**

Single-cell RNA sequencing (scRNA-seq) technology enables biological research at single-cell resolution. Cell clustering is a crucial task in scRNA-seq data analysis since it provides insights into cell heterogeneity. Although existing methods have made significant progress in this task, it remains challenging to fully utilize the relationship among cells.

**Results:**

We propose Interpretable Graph Contrastive Learning method with Adaptive Positive Sampling (IGCLAPS), a novel end-to-end graph contrastive clustering method for scRNA-seq data analysis. Specifically, IGCLAPS learns low-dimensional embeddings with a graph transformer, based on which a dual-head graph contrastive learning module is used to perform dimension reduction and cell clustering simultaneously. Besides, an accurate definition of positive sample pairs is crucial in contrastive learning, we devise an adaptive positive sampling module, which dynamically identifies true positive sample pairs based on both expression similarity and soft cluster labels generated by the contrastive learning module. Extensive experiments on a series of real datasets including cell clustering, visualization, and differential expression analysis demonstrate that IGCLAPS can effectively enhance clustering performance and generate interpretable gene expression patterns of scRNA-seq data.

**Availability and implementation:**

The source codes of IGCLAPS are available at https://github.com/ZhengWeihuaYNU/IGCLAPS.

## Graphical abstract

**Figure:**
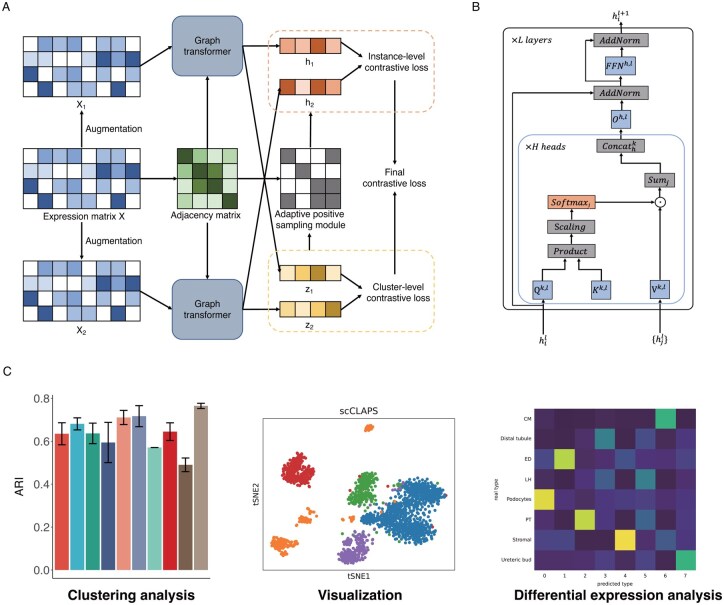

## 1 Introduction

The past decade has witnessed the rapid development of single-cell RNA sequencing (scRNA-seq) technology, which provides transcriptomic profiles of individual cells and hence allows us to study expression patterns at single-cell resolution. One of the crucial tasks in scRNA-seq analysis is cell clustering, since it divides cells into distinct subpopulations and hence offers insights of heterogeneity between different cell groups, based on which other downstream tasks such as differential expression analysis and cell-cell communication inference can be performed (Stegle *et al.* 2015, Kiselev *et al.* 2019, Andrews *et al.* 2021). However, clustering analysis of scRNA-seq data is hindered by its sparsity, prevalent noise, and extremely high dimensionality (Lähnemann *et al.* 2020, Zhang *et al.* 2021, Kharchenko 2021, Wang *et al.* 2022), and effective clustering methods for scRNA-seq data are still imperative.

A large amount of computational methods for cell clustering have been proposed from different perspectives in the past few years. Early studies on single-cell data clustering mainly make use of traditional statistical or machine learning methods. For instance, pcaReduce (Žurauskienė and Yau 2016) applied PCA to expression data and used the top components for subsequent clustering, while SC3 applied PCA to the distance matrix and performed consensus clustering by clustering cells for multiple times (Kiselev *et al.* 2017). CIDR (Lin *et al.* 2017) conducted principal coordinate analysis on the dissimilarity matrix of the gene expression and performed hierarchical clustering on the principal components, and ZIFA (Pierson and Yau 2015) adopted a modified probabilistic PCA method incorporating a zero-inflated distribution for dimensionality reduction and clustering. These methods typically involve statistical modeling, which may pose limitations on their applicability. In recent years, deep learning methods have also been applied to the analysis of scRNA-seq data since they do not require any statistical assumptions and are able to learn complex patterns. One main type of deep learning-based clustering methods for scRNA-seq data is based on auto-encoders. For instance, scDeepCluster (Tian *et al.* 2019) used a deep count auto-encoder to perform dimension reduction and cell clustering by minimizing the zero-inflated negative binomial loss and the clustering loss simultaneously. MoE-Sim-VAE (Kopf *et al.* 2021) used a variational auto-encoder (VAE) with a Mixture of Experts (MoE) architecture to learn various modes from the data. scGNN (Wang *et al.* 2021) combined graph neural network-based auto-encoder with a left-truncated mixture Gaussian model to learn gene expression patterns. Similarly, scGAC (Cheng and Ma 2022) combined graph attentional auto-encoder with KL divergence to perform representation learning and cell clustering at the same time. Recently, scMAE (Fang *et al.* 2024), which combined masked auto-encoder with a masked predictor, was proposed to learn low-dimensional embeddings of scRNA-seq data. Apart from methods based on auto-encoders, contrastive learning-based methods have also been investigated in dimension reduction and clustering of scRNA-seq data. The main idea of contrastive learning is to project positive augmented pairs of the original data (i.e. augmentations generated from the same instance) closely in the low-dimensional representation space and push apart negative pairs. One of the earliest contrastive learning-based clustering methods for scRNA-seq data is contrastive-sc (Ciortan and Defrance 2021), which used instance-level contrastive loss for representation learning. CLEAR (Han *et al.* 2022) used a novel data augmentation method for contrastive learning. scCCL (Du *et al.* 2023) used both instance-level and cluster-level contrastive loss to perform dimension reduction and clustering in an end-to-end manner. Similarly, scDCCA (Wang *et al.* 2023) integrated both auto-encoder and contrastive learning to jointly perform representation learning and cell clustering. Recently, graph neural networks have also been used in contrastive learning methods for scRNA-seq data clustering. For instance, scGCC (Tian *et al.* 2023) used graph attention network and designed several augmentation strategies, while scGPCL (Lee *et al.* 2023) focused on increasing the number of positive pairs by pulling together an anchor cell with its cluster prototype.

The aforementioned methods have made great progress in single-cell clustering, yet there still remain some limitations that impede further improvement of the clustering performance. First, precisely defining positive pairs is one of the most important steps in contrastive learning (Shen *et al.* 2023). However, it has been pointed out (Wan *et al.* 2022, Lee *et al.* 2023) that most existing single-cell clustering methods based on contrastive learning either generate only one positive pair for each cell, which do not sufficiently exploit the information of homogeneous cells, or simply use cells with similar expression as positive pairs, which may introduce extra noise generated by false positive pairs. Therefore, methods that can accurately identify homogeneous cells as positive pairs are highly desirable. Second, single-cell clustering methods based on auto-encoders or contrastive learning depend on black-box deep learning networks; hence, interpretable methods that can provide intuitive biological insights are highly favorable. Moreover, although there have been some contrastive clustering methods that use an additional cluster head to generate cell clustering results, most of them aim to minimize the cluster-level contrastive loss and do not make full use of the soft-label representations generated by the cluster head.

To address the issues mentioned above, we propose IGCLAPS, a novel end-to-end deep clustering method based on graph contrastive learning. IGCLAPS first constructs the K-nearest neighbor (KNN) graph according to cosine distance among cells. Then, it uses graph transformer, which is a powerful graph deep learning model, to learn low-dimensional embeddings of the data. The embeddings are then projected into two different representation spaces, in which the instance-level and cluster-level contrastive loss are calculated, respectively. To more accurately define positive and negative sample pairs, we devise an adaptive positive sampling module, which uses the cluster representations combined with neighbor cells found by KNN graph to identify multiple positive pairs, based on which the final contrastive loss is calculated. Experiments including cell clustering, t-SNE visualization, and differential expression analysis on various real scRNA-seq datasets demonstrate the superior performance and interpretability of IGCLAPS. In addition, ablation studies are also conducted to show the significance of all modules in IGCLAPS.

## 2 Materials and methods

**Figure 1.**
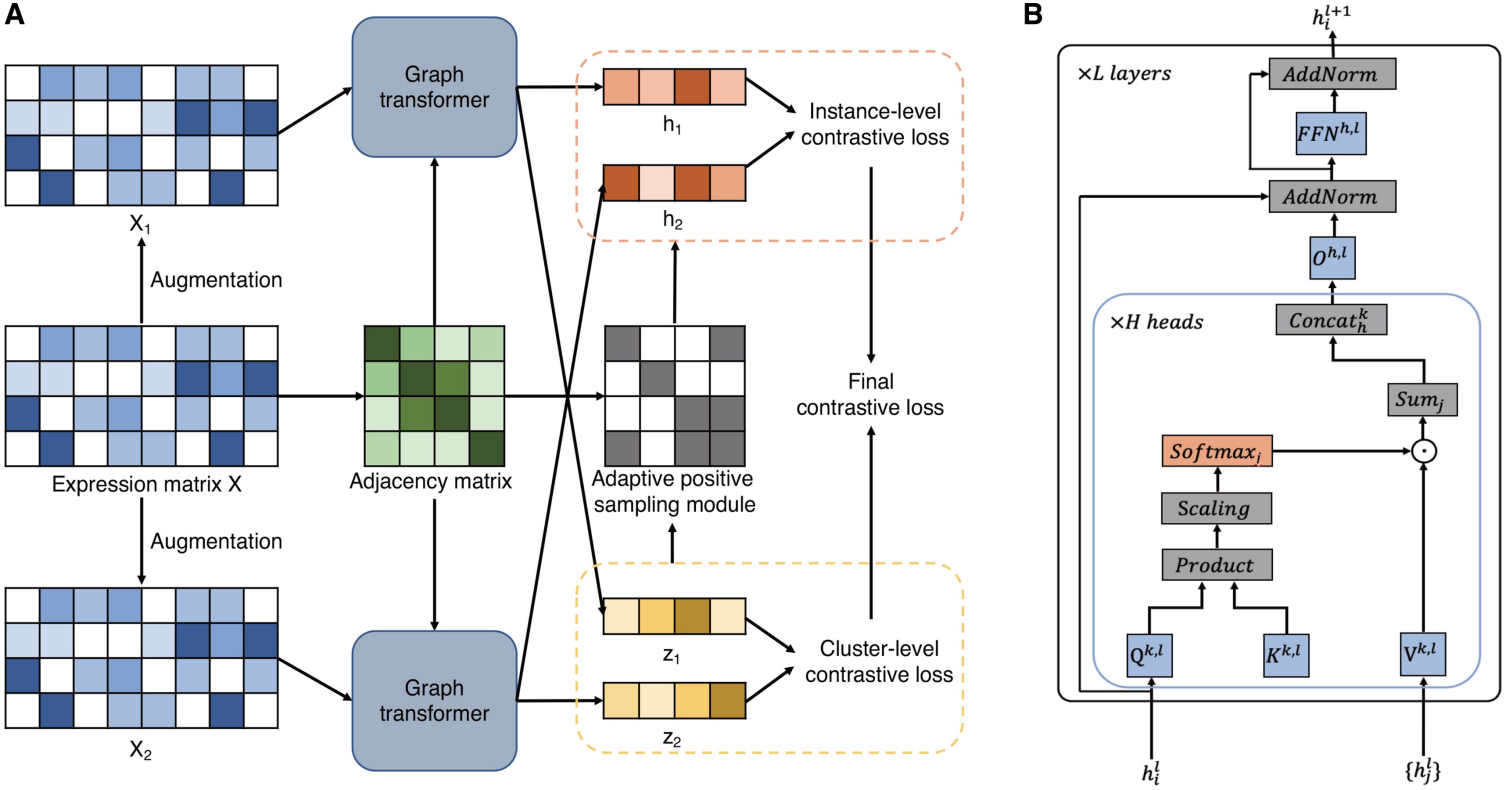
The framework of IGCLAPS. (A) Flowchart of IGCLAPS. (B) Architecture of graph transformer used in IGCLAPS.

The framework of IGCLAPS is shown in Fig. 1. IGCLAPS is comprised mainly of four parts, i.e. data augmentation, graph transformer network, dual-head graph contrastive learning module, and adaptive positive sampling module. Specifically, consider a raw-count expression matrix $X_{N\times P}$ with *N* cells and *P* genes. After data preprocessing (e.g. quality control, log-transformation, and highly variable genes selection), the data will be transformed into $X_{n\times p}$ consisting of *n* cells and *p* genes, based on which the KNN graph *G* and its corresponding adjacency matrix $A_{n\times n}$ are constructed according to cosine distance. The normalized gene expression matrix is then used to generate two augmented views $X^{\left(1\right)}$ and $X^{\left(2\right)}$ by randomly masking a proportion of the gene expression. $X^{\left(1\right)}$, $X^{\left(2\right)}$ and *A* are then fed into two identical graph transformers $G_{1}$ and $G_{2}$ to yield low-dimensional embeddings $Z^{\left(1\right)}$ and $Z^{\left(2\right)}$. These two embeddings are further put into an instance-level contrastive head and a cluster-level contrastive head to generate two latent representations, based on which the instance-level and cluster-level contrastive loss are calculated, and the representations from the cluster-level contrastive head are also used as the soft cluster labels. Besides, to accurately define positive samples and make full use of information carried by the cluster head, IGCLAPS adopts an adaptive positive sampling (APS) module, which uses both the cluster head and adjacency matrix to dynamically define positive sample pairs, and the cluster-level loss and instance-level loss modified by APS comprise the final loss.

### 2.1 Data preprocessing and augmentation

In this article, the raw-count matrix of scRNA-seq data with cells as rows and genes as columns is preprocessed with the following steps: First, we perform quality control on the datasets, which removes cells expressing <5 genes and genes expressed in <5 cells. Second, we normalize the data with a log-transformation. Finally, we retain 3000 highly variable genes to include the most useful biological information and avoid possible noises in those genes with less variability. The preprocessing steps in this article are performed with the widely used Python package SCANPY (Wolf *et al.* 2018).

A crucial step of contrastive learning is to generate two different augmentations of the same instance, as the augmentations from the same instance will be regarded as a positive pair, while other pairs will be treated as negative pairs. In this study, we adopt a simple but effective data augmentation scheme (Gao *et al.* 2021): consider a cell expression vector $X_{i}$, two augmented views denoted by $X_{i}^{\left(1\right)}$ and $X_{i}^{\left(2\right)}$ are generated by randomly masking some gene expression of $X_{i}$. Here, we randomly mask 50% of the gene expression for augmentation, while the adjacency graph is not perturbed since improper perturbation may destroy the graph structure and hence deteriorate the clustering performance (Yu *et al.* 2022, Kim *et al.* 2022). The augmented views $X^{\left(1\right)}$ and $X^{\left(2\right)}$ generated from the original normalized expression matrix **X** along with the adjacency matrix *A* are then fed into the graph transformer networks to yield low-dimensional embeddings.

### 2.2 Graph transformer network

Graph transformer is a generalization of transformer to graphs (Dwivedi and Bresson 2021), which takes into consideration the adjacency relationships between instances to learn low-dimensional embeddings. The core part of a graph transformer layer is the multi-head graph attention mechanism: Denote the embedding of $X_{i}$ at the $l_{\text{th}}$ layer as $h_{i}^{l}$, and its corresponding adjacency matrix as *A*, the embedding at the ${\left(l+1\right)}_{\text{th}}$ layer can be formulated as:

(1) $${\hat{h}}_{i}^{l+1}=O_{h}^{l}|{|}_{k=1}^{H}(\sum_{j\in N_{i}}w_{ij}^{k,l}V^{k,l}h_{j}^{l}),$$

where $w_{ij}^{k,l}$ is the weight which can be calculated by $w_{ij}^{k,l}=\mathrm{softma}x_{j}(\frac{Q^{k,l}h_{i}^{l}\cdot K^{k,l}h_{j}^{l}}{\sqrt{d_{k}}})$, *H* is the number of attention heads, $d_{k}=d/H$ is the output dimension of each head, ‖ is the concatenation operation, $N_{i}$ is the set of neighbors of cell *i* with $A_{ij}=1$, and $Q^{k,l},K^{k,l},V^{k,l}\in R^{d_{k}\times d},O_{h}^{l}\in R^{d\times d}$ are learnable weight matrices. The output embedding ${\hat{h}}_{i}^{l+1}$ is then passed to a fully connected feed-forward layer (FFN), followed by residual connections and normalization layers. In summary, the output of the graph transformer layer can be represented by:

(2) $${\tilde{h}}_{i}^{l+1}=\mathrm{Norm}(h_{i}^{l}+{\hat{h}}_{i}^{l+1}),$$

 

(3) $${\bar{h}}_{i}^{l+1}=W_{2}^{l}R\mathrm{eLU}(W_{1}^{l}{\tilde{h}}_{i}^{l+1}),$$

 

(4) $$h_{i}^{l+1}=\mathrm{Norm}({\tilde{h}}_{i}^{l+1}+{\bar{h}}_{i}^{l+1}),$$

where Norm(·) is the normalization operation, ReLU(·) is the ReLU activation function, $W_{1}^{l}\in R^{2d\times d}$, $W_{2}^{l}\in R^{d\times 2d}$ are learnable parameters, ${\tilde{h}}_{i}^{l+1}$ and ${\bar{h}}_{i}^{l+1}$ are the intermediate representations and $h_{i}^{l+1}$ denotes the final output of the transformer layer. In IGCLAPS, we simply use a dropout layer to perform data augmentation, and the two augmented views $X^{\left(1\right)}$ and $X^{\left(2\right)}$ are then encoded by two graph transformers with identical structure. The embeddings learned by graph transformers are denoted as $h^{\left(1\right)}$ and $h^{\left(2\right)}$.

### 2.3 Dual-head graph contrastive learning module

To capture both instance-level and cluster-level information of the data, we adopt the dual-head graph contrastive learning scheme. Given $h^{\left(1\right)}$ and $h^{\left(2\right)}$ learned by graph transformers, we use a multi-layer perceptron (MLP) as the instance-level contrastive head and project $h^{\left(1\right)}$ and $h^{\left(2\right)}$ into a latent space. Specifically, denote the embeddings learned from two views through the instance-level contrastive head by $z^{\left(1\right)}=\{z_{1}^{\left(1\right)},\ldots ,z_{N}^{\left(1\right)}\}$ and $z^{\left(2\right)}=\{z_{1}^{\left(2\right)},\ldots ,z_{N}^{\left(2\right)}\}$, i=1,…,N. Selecting $z_{i}^{\left(1\right)}$ as the anchor, traditional contrastive learning methods use $z_{i}^{\left(2\right)}$ as the only positive sample of the $z_{i}^{\left(1\right)}$. In comparison, we use the neighbor contrastive loss (Shen *et al.* 2023), which regards neighbor cells from both views, i.e. $z_{j}^{\left(1\right)}$ and $z_{j}^{\left(2\right)},A_{ij}=1$, as positive samples. Specifically, neighbor contrastive loss of $z_{i}^{\left(1\right)}$ takes the form

(5) $$l_{i}^{\left(1\right)}=-\log\frac{e^{s(z_{i}^{\left(1\right)},z_{i}^{\left(2\right)})/\tau }+\sum_{\left\{j|A_{ij}=1\right\}}\left(e^{s(z_{i}^{\left(1\right)},z_{j}^{\left(1\right)})/\tau }+e^{s(z_{i}^{\left(1\right)},z_{j}^{\left(2\right)})/\tau }\right)}{\sum_{k=1}^{N}\left(e^{s(z_{i}^{\left(1\right)},z_{k}^{\left(1\right)})/\tau }+e^{s(z_{i}^{\left(1\right)},z_{k}^{\left(2\right)})/\tau }\right)},$$

in which τ is the temperature parameter, s(·) is cosine similarity. Considering instances from both views, the instance-level graph contrastive loss is formulated as:

(6) $$L_{\mathrm{ins}}=\frac{1}{2N}\sum_{i=1}^{N}\left(l_{i}^{\left(1\right)}+l_{i}^{\left(2\right)}\right).$$

Similar to the instance-level contrastive head, we also use MLP as the cluster-level contrastive head, which projects $h^{\left(1\right)}$ and $h^{\left(2\right)}$ into an *M*-dimension latent space, where *M* is the number of clusters. Given $Y^{\left(1\right)}$ as the embeddings of the first view in this space, we use the column vectors of $Y^{\left(1\right)}$, denoted by $y_{1}^{\left(1\right)},\ldots ,y_{M}^{\left(1\right)}$ as the representations of the *M* clusters, based on which we use the assignment graph contrastive loss (Zhong *et al.* 2021) as cluster-level contrastive loss. The cluster-level contrastive loss of $y_{i}^{\left(1\right)}$ is defined as:

(7) $${\hat{l}}_{i}^{\left(1\right)}=-\log\frac{e^{s(y_{i}^{\left(1\right)},y_{i}^{\left(2\right)})/\tau }}{\sum_{j=1}^{M}[e^{s(y_{i}^{\left(1\right)},y_{j}^{\left(1\right)})/\tau }+e^{s(y_{i}^{\left(1\right)},y_{j}^{\left(2\right)})/\tau }]},i=1,\ldots ,M.$$

To avoid assigning most instances to a single cluster, we use a cluster regularization that is formulated as:

(8) $$L_{\text{reg}}=\log(M)-H(Y),$$

where

(9) $$H(Y)=-\sum_{i=1}^{M}\sum_{v=1}^{2}P(Y_{i}^{\left(v\right)})\mathrm{logP}(Y_{i}^{\left(v\right)}),$$

 

(10) $$P(Y_{i}^{\left(v\right)})=\frac{\sum_{j=1}^{N}Y_{ji}^{\left(v\right)}}{\sum_{j=1}^{N}\sum_{i=1}^{M}Y_{ji}^{\left(v\right)}},$$

and the final cluster-level contrastive loss is

(11) $$L_{\mathrm{cls}}=\frac{1}{2M}\sum_{i=1}^{M}\left({\hat{l}}_{i}^{\left(1\right)}+{\hat{l}}_{i}^{\left(2\right)}\right)+L_{\mathrm{reg}}.$$

### 2.4 Adaptive positive sampling module

The instance-level contrastive loss in Equation (5) aims to map cells with similar expression levels closely in the latent space. However, since cells with similar expression profiles may belong to different cell types, mapping these cells closely will deteriorate the performance of dimension reduction and clustering. Therefore, we propose an APS module, which makes use of the soft-label information carried by the cluster-level contrastive head to adaptively define positive samples. Specifically, given the embeddings $Y^{\left(1\right)}$ and $Y^{\left(2\right)}$ generated by the cluster-level contrastive head, we denote the row vectors by $g_{i}^{\left(v\right)},i=1,\ldots ,N,v=1,2$, then the smoothed representations used for cluster assignments are formulated as

(12) $$g_{i}=A[(g_{i}^{\left(1\right)}+g_{i}^{\left(2\right)})/2],$$

which can be regarded as soft cluster labels of the cells. Then we calculate the soft-label guided adjacency matrix $\hat{A}$ with its elements defined as:

(13) $${\hat{A}}_{ij}=\{\begin{array}{cc} 1, & \text{if}\;s(g_{i},g_{j})\geq \lambda ,\\ 0, & \text{otherwise}. \end{array}$$

where s(·) is the cosine similarity and λ is the similarity threshold. Combined with the adjacency matrix *A* based on the KNN graph, it is now possible to define the final adjacency matrix as

(14) $$A^{\mathrm{final}}=A⊙\hat{A}.$$

Given $A^{\text{final}}$ which jointly considers gene expression similarity and soft-label similarity between cells, only cells with both similar expression levels and soft-labels will be regarded as neighbor cells, and augmentations of these cells will be recognized as positive pairs. Then the modified instance-level contrastive loss can be calculated by replacing *A* with $A^{\text{final}}$ in Equation (5), and the final loss of IGCLAPS is formulated as:

(15) $$L=\alpha L_{\mathrm{ins}}+(1-\alpha )L_{\mathrm{cls}},\;\alpha \in [0,1],$$

where α is a weight parameter.

## 3 Results

### 3.1 Parameter settings

Before conducting numerical experiments, we first summarize the parameter settings of IGCLAPS. IGCLAPS is implemented in Python using PyTorch. In the data augmentation stage, the default dropout rate is set to 0.5. We use a single multi-head attention layer with four heads in graph transformer, while the dimension of the embeddings generated by the graph transformer is 32, and the number of maximum training epochs is set to 500. The initial learning rate is set to 0.001. To accelerate the training process and avoid large computational cost, we use the Python library torch_geometric to divide graph data into mini-batches. The batch size used in mini-batch changes according to the size of the datasets. For datasets with <1000 cells, the batch size is set to 64, while it is set to 256 for datasets containing 1000–2000 cells. For datasets with 2000–10 000 cells, the batch size is set as 512, and for large datasets with more than 10 000 cells, the batch size is set to 1024. In the contrastive loss, the temperature parameter τ and the weight parameter are both set to 0.5. As for the number of neighbors in KNN graph construction denoted by *k*, we select the optimal value of it by cross-validation since a feasible *k* changes according to both sample sizes and number of cell types in the datasets. It is also possible for the users of IGCLAPS to determine optimal hyper-parameters by minimizing the final contrastive loss without knowing the real cell type labels. All experiments are implemented with an NVIDIA RTX 4090D GPU (24 GB).

### 3.2 Clustering performance

**Figure 2.**
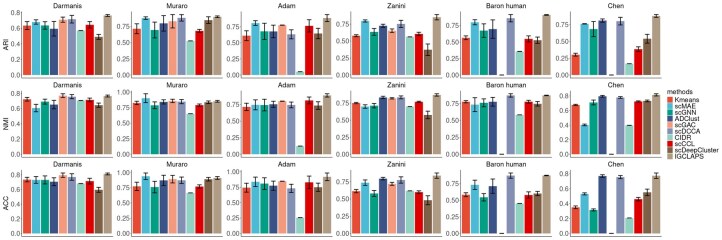
Clustering results of different methods. Higher values indicate better results.

We now assess the performance of IGCLAPS in cell clustering. Twelve real scRNA-seq datasets (Darmanis *et al.* 2015, Baron *et al.* 2016, La Manno *et al.* 2016, Muraro *et al.* 2016, Adam *et al.* 2017, Chen *et al.* 2017, Zheng *et al.* 2017, Han *et al.* 2018, Young *et al.* 2018, Colquitt *et al.* 2021, Zanini *et al.* 2023) are used as benchmark datasets. These datasets involve different species and tissues with sample sizes ranging from <500 to >10 000. Detailed description and availability of these datasets can be found in Table 1, available as supplementary data at *Bioinformatics* online. To assess the clustering performance, nine widely used clustering methods, namely KMeans, CIDR (Lin *et al.* 2017), ADClust (Zeng *et al.* 2022), scDeepCluster (Tian *et al.* 2019), scGNN (Wang *et al.* 2021), scMAE (Fang *et al.* 2024), scGAC (Cheng and Ma 2022), scCCL (Du *et al.* 2023), and scDCCA (Wang *et al.* 2023), which include statistical methods, auto-encoder based methods, and contrastive learning-based methods, are compared with IGCLAPS. Availability of these methods can be found in Table 2, available as supplementary data at *Bioinformatics* online. Adjusted Rand index (ARI) (Hubert and Arabie 1985), normalized mutual information (NMI) (Strehl and Ghosh 2002), and accuracy (ACC) are used as the evaluation metrics. As can be seen in Fig. 2 and Fig. 1, available as supplementary data at *Bioinformatics* online, in 9 of 12 datasets, IGCLAPS achieves the highest ARI among all ten methods compared in this article (note that scGAC is unable to deal with Baron human and Chen data, which contain more than 8000 cells with the device used in this article). In the Bladder data, IGCLAPS takes the second place, while in La Manno and Young data, it takes the third place. The overall performance of IGCLAPS in terms of NMI is similar: IGCLAPS achieves the highest NMI in 9 of 12 datasets. In Darmanis data, scGAC has the highest NMI, followed by IGCLAPS, while in Muraro and Baron mouse data, IGCLAPS takes the third place. As for ACC, IGCLAPS achieved best performance in seven of 12 datasets and is among the top three methods in all datasets except Baron mouse, in which ADClust, scMAE, and scDCCA had higher accuracy than IGCLAPS. Nevertheless, IGCLAPS outperforms its competitors in most cases, and it is noteworthy that although deep learning methods achieve better results than traditional statistical methods, i.e. CIDR and KMeans in most datasets, IGCLAPS is the only method that consistently outperforms CIDR and KMeans in all cases. Overall, through evaluation with ARI, NMI, and ACC, it is apparent that IGCLAPS is able to identify cell subpopulations accurately.

**Table 1.** Results of ablation test comparing IGCLAPS and the ablated models.^a^

Neighbors	Cluster-head	APS	Darmanis	La Manno	Muraro	Bladder	Adam	Zanini	Colquitt	PBMC	Young	Baron-h	Baron-m	Chen
✓	✓	✓	**0.765**	**0.576**	**0.900**	**0.680**	**0.886**	**0.852**	**0.860**	**0.782**	**0.692**	**0.912**	0.842	**0.898**
✓	✓	×	0.676	0.554	0.886	0.629	0.833	0.813	0.850	0.756	0.684	0.899	**0.859**	0.868
×	✓	×	0.711	0.497	0.880	0.588	0.770	0.776	0.734	0.581	0.646	0.711	0.825	0.775
✓	×	×	0.748	0.469	0.787	0.549	0.794	0.682	0.818	0.768	0.655	0.746	0.549	0.553

a ARI is used as an evaluation metric. Best results are marked in bold.

### 3.3 t-SNE visualization

**Figure 3.**
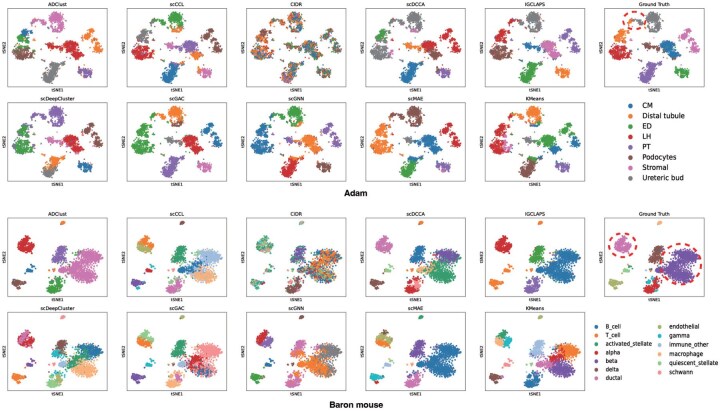
t-SNE visualization of different methods on Adam and Baron mouse dataset. The legends correspond to the ground truth results.

After evaluating the clustering performance of IGCLAPS with ARI and NMI, we now visualize the clustering results of IGCLAPS with t-SNE (Maaten and Hinton 2008) to investigate its performance more intuitively. The visualization results of different methods on Adam and Baron mouse data are shown in Fig. 3, and t-SNE results of other datasets can be found in Fig. 2, available as supplementary data at *Bioinformatics* online. It is obvious that IGCLAPS is able to divide cells into the ground truth subpopulations more precisely. For instance, in Adam data, IGCLAPS is the only method that accurately identifies ureteric buds and distal tubules. In the Baron mouse dataset, compared with IGCLAPS, most methods fail to recognize beta cells as a single subpopulation but divide it into several groups in different manners. Similarly, IGCLAPS is able to identify ductal cells while most other methods falsely split them into different groups. Given the results of other datasets, we can see that IGCLAPS correctly identifies large subpopulations in the data instead of falsely dividing them into multiple small groups, indicating that our method effectively utilizes the information of neighbor cells. In summary, the t-SNE visualization results clearly illustrate that IGCLAPS is able to significantly improve clustering results and divide cells into their subpopulations accurately, demonstrating that IGCLAPS effectively exploits the biological information in the data.

### 3.4 Differential expression analysis

**Figure 4.**
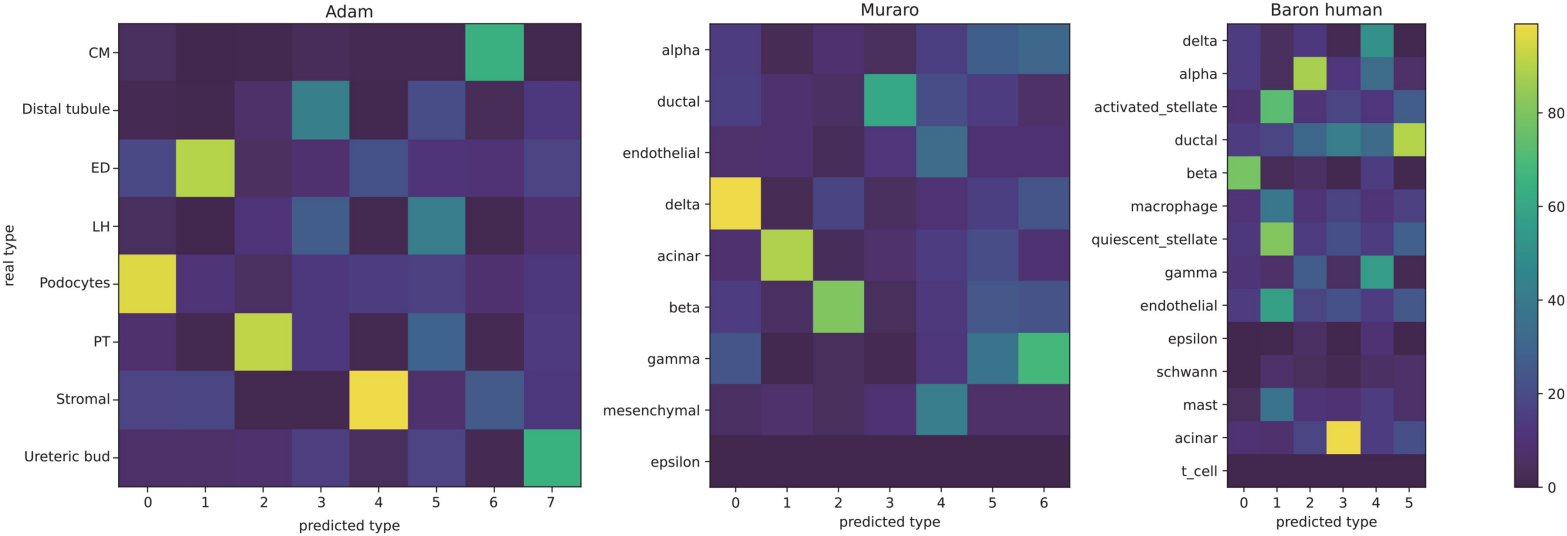
Heatmap of the overlapped DEGs found by Seurat and IGCLAPS.

Differential expression analysis is one of the main downstream tasks of scRNA-seq data analysis, which aims to define the sets of differentially expressed genes (DEGs) that can best discriminate different subpopulations of cells. In this section, we perform differential expression analysis on the datasets used in the clustering analysis to investigate whether IGCLAPS is able to find DEGs in the data. Besides, since IGCLAPS performs dimension reduction and clustering in a one-stop manner, it is possible to interpret the clustering results by calculating the contribution of different genes in determining the cluster assignment of each cell. To illustrate the interpretability of IGCLAPS, we use the integrated gradients method (Sundararajan *et al.* 2017) to visualize the genes that contribute to the cluster assignments of different cells and compare them to the DEGs found by Seurat (Butler *et al.* 2018), which is a commonly used R package for scRNA-seq data analysis. More specifically, we first use Seurat to generate the top 200 DEGs of each real cell type with the maximum false discovery rate of 0.01 and minimum log fold-change of 1, then we calculate the integrated gradients contributed by different genes of each predicted cell type and select the top 200 genes with the largest integrated gradients. The number of overlapped genes between DEGs and genes identified by IGCLAPS in Adam, Muraro, and Baron human data is shown in Fig. 4, and results of other data can be found in Fig. 3, available as supplementary data at *Bioinformatics* online. It can be seen that for those datasets with the best clustering performance, all subpopulations identified by IGCLAPS correspond to some true cell types. For instance, in the Adam data, cell type 4 predicted by IGCLAPS is highly similar to stromal cells in terms of DEGs, and the predicted cell type 0 in the Muraro data obviously corresponds to delta cells. It can be seen that the number of cell types identified by IGCLAPS is not equal to the number of true cell types in some cases, and the reasons can also be explained by this experiment. For example, in the Baron human data, the epsilon, Schwann, and T cells do not correspond to any types predicted by IGCLAPS in terms of DEGs, and the reason is that there are only 3 epsilon cells, 7 T cells, and 13 Schwann cells in this dataset, which contains more than 8000 cells in total. Nevertheless, it is apparent that IGCLAPS is able to discover the expression patterns of different cell types and generate interpretable results.

**Figure 5.**
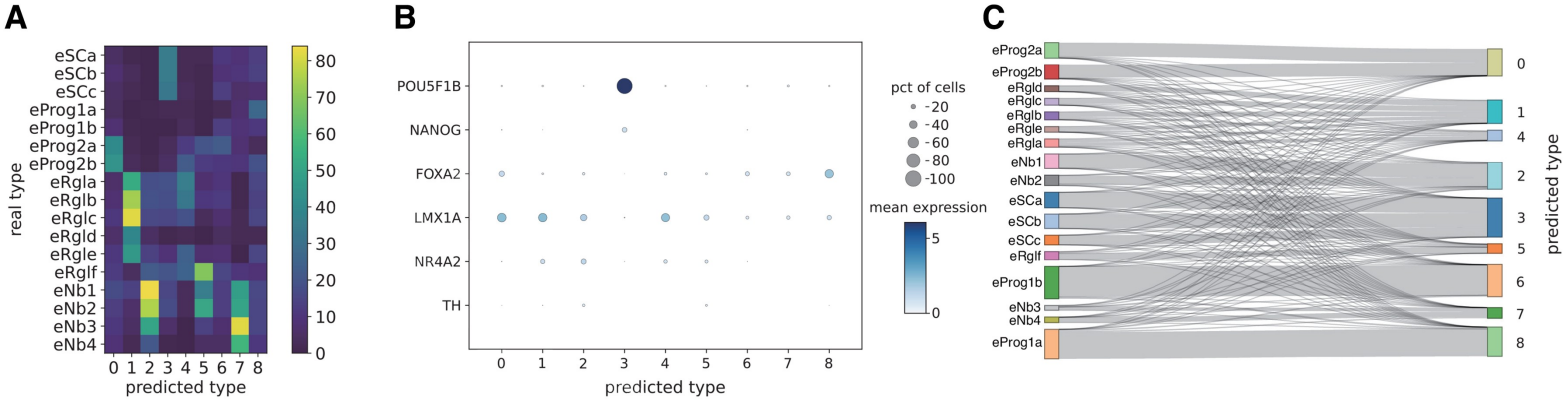
IGCLAPS generates interpretable analysis results on La Manno data. (A) Overlapped DEGs found by Seurat and integrated gradients. (B) Dotplot of some marker genes found in the predicted types. (C) Sanky plot between real cell types and predicted types.

To further demonstrate the interpretability of IGCLAPS, we focus on the La Manno dataset, which is a highly complicated dataset of human embryonic stem cells with numerous refined cell subpopulations defined through biological experiments (La Manno *et al.* 2016). It can be seen in Figs 1 and 2, available as supplementary data at *Bioinformatics* online that none of the methods compared in this article can achieve ideal clustering performance on this dataset in terms of ARI and NMI, and we now investigate whether IGCLAPS is able to generate informative and interpretable results on this dataset. We first inspect the heatmap depicting overlapped DEGs found by Seurat and integrated gradients. As Fig. 5A shows, most of the cell clusters found by IGCLAPS contain DEGs from multiple real cell types. However, it is notable that in most cases, the DEGs of cell types defined by IGCLAPS correspond to several related real cell types. For instance, cluster 1 contains DEGs of radial glia-like cells (eRgla-e), while cluster 7 corresponds to neuroblast (eNb1-4), indicating that IGCLAPS may recognize biologically related real cell subpopulations and divide them into the same cluster. A dotplot of important marker genes found in the original research of La Manno data is shown in Fig. 5B. It can be seen that POU5F1B and NANOG are expressed only in cluster 3, LMX1A has relatively high expression levels in clusters 0, 1, 2, and 4, FOXA2 is more abundant in clusters 0 and 8 than other clusters, while TH and NR4A2 are not found in these two clusters. According to the original research, these expression patterns indicate that cluster 3 consists of stem cells, while clusters 0 and 8 may be comprised of progenitors. The Sanky plot shown in Fig. 5C clearly validates our assumption, and the results show that IGCLAPS identifies expression patterns shared by cells with common parental cell types, indicating that it is able to extract information among different cell types and provide interpretable biological insights even when the dataset is highly complex.

### 3.5 Ablation studies and robustness test

As we have demonstrated before, IGCLAPS is a deep clustering method consisting of multiple modules, i.e. cluster-level contrastive head, neighborhood-based instance-level contrastive head, and the APS module, which aims to accurately distinguish real positive sample pairs from those that have similar expression levels but belong to different cell types. In this section, we perform an ablation experiment to manifest the effectiveness and necessity of these modules. Specifically, we investigate three ablated models: in the first ablated model, we remove the APS module proposed in this article, hence the model regard all neighbor cells as positive pairs instead of further identifying highly confident positive pairs from these neighbor cells. In the second ablated model, we remove the adjacency matrix derived from the gene expression similarity between cells, which turns the model into an ordinary contrastive clustering model using only one positive pair for each anchor cell. In the last ablation scheme, both the APS module and cluster head are ablated, leading to a model that uses all neighbor cells as positive pairs and generates only the low-dimensional embeddings without cluster assignments. We compare the clustering results of these ablated models to those of IGCLAPS. The 10-time average clustering results measured by ARI are shown in Table 1, and results in terms of NMI are shown in Table 3, available as supplementary data at *Bioinformatics* online. Apparently, removing any part of IGCLAPS deteriorates the clustering results in most cases, demonstrating the necessity of all modules in IGCLAPS.

**Figure 6.**
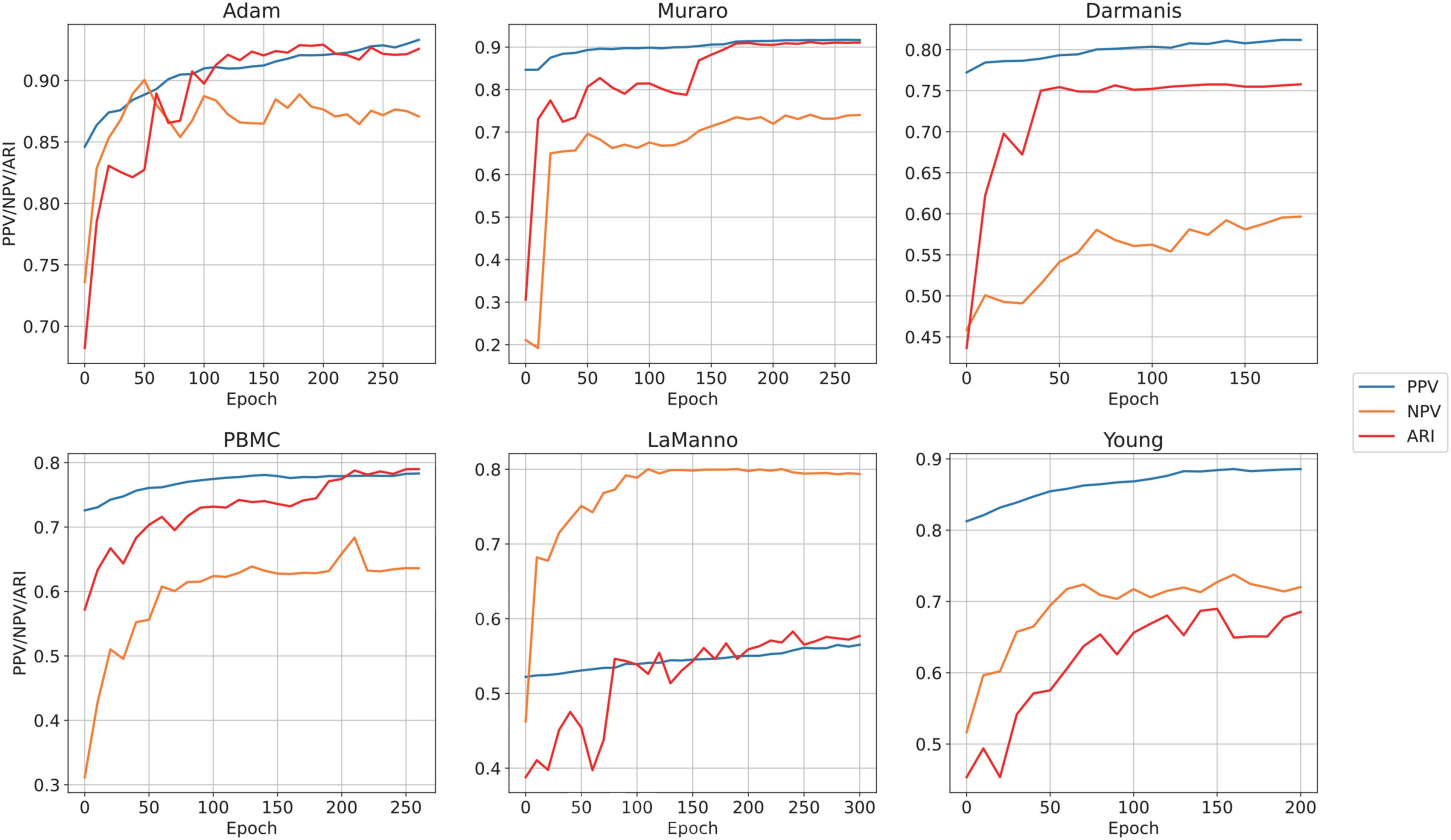
PPV and NPV of positive and negative samples identified by IGCLAPS.

Through the ablation study shown above, it is obvious that our model, which aims to more accurately define positive and negative sample pairs in graph contrastive learning, is able to improve the clustering results of scRNA-seq data. To display the efficacy of our model more straightforwardly, we now investigate how it divides positive and negative sample pairs during the training process. Specifically, we calculate the positive predicted values (PPV) and negative predicted values (NPV) of the sample partition, which measure the proportion of truly identified positive and negative pairs, respectively. Besides, we draw the ARI curve to see how the clustering performance changes along with PPV and NPV. According to the results shown in Fig. 6, both the PPV and NPV gradually rise with the increase of ARI, indicating that the APS module helps identify cells of the same cell type among cells with similar expression values more accurately, which also explains the reasons why IGCLAPS is able to improve the clustering performance.

We then test the robustness of IGCLAPS through changing two main parameters, i.e. masking proportion in the data augmentation stage and number of clusters. Specifically, we first test the clustering performance of IGCLAPS with the masking proportion ranging from 0.5 to 0.9. The results are shown in Table 4, available as supplementary data at *Bioinformatics* online. It can be seen that IGCLAPS is robust to changes of masking proportion; only when it grows to an extreme level, e.g. 90%, does it have an obvious influence on the clustering performance. We then test the performance of IGCLAPS when the exact number of ground truth cell types is unknown. Specifically, we use silhouette coefficients (Rousseeuw 1987) to determine the number of clusters in an unsupervised manner and calculate ARI and NMI (ACC is not applicable in this case as the number of clusters is not equal to that of real cell types). The results are shown in Table 5, available as supplementary data at *Bioinformatics* online. It can be seen that in most cases, except Darmanis and Adam data, IGCLAPS achieves robust clustering performance even when the precise number of cell types is unknown. In Zanini data, the clustering results are even better when the number of ground truth cell types is unknown. To sum up, IGCLAPS shows ideal robustness to variation of parameters such as masking proportion in data augmentation and number of clusters.

## 4 Conclusion

In this article, we propose a novel method named IGCLAPS for scRNA-seq data analysis, which is based on graph contrastive clustering with an APS module to accurately define true positive pairs among all neighbor cells. We demonstrated the effectiveness and interpretability of IGCLAPS through extensive experiments including cell clustering, visualization, and differential expression analysis on a series of real datasets. Besides, to validate the necessity of the modules in IGCLAPS, we conducted ablation experiments by removing different components of IGCLAPS, and the results proved the necessity of all modules. Besides, we calculated the PPV and NPV of positive and negative pairs recognized by IGCLAPS to demonstrate that the APS module helps define positive pairs more precisely, which finally improves the clustering performance. Furthermore, we tested the performance of IGCLAPS by changing the masking proportion in data augmentation and the number of clusters, and the results showed that IGCLAPS is robust to variation of parameters.

Although IGCLAPS effectively utilizes graph contrastive clustering methods combined with an APS module to enhance the performance of single-cell clustering, it is still possible to make further improvements in several aspects. For instance, it is challenging to determine the optimal number of neighbors in KNN graph construction as it is influenced by the size of the dataset and the number of subpopulations in it. Hence, methods that can adaptively construct KNN graphs of different radii are desirable in graph contrastive learning. Besides, since data augmentation is a preliminary step in contrastive learning and has a direct impact on the performance of the model, combining biological information with data augmentation strategies will be a valuable research topic. Moreover, data from other omics, such as spatial transcriptomics or chromatin accessibility, may provide extra information from different perspectives and generate more accurate and biologically meaningful results.

## Supplementary Material

btaf411_Supplementary_Data

## Data Availability

The datasets underlying this article are available at https://github.com/ZhengWeihuaYNU/IGCLAPS.
