# Supplementary material for: IGCLAPS: an interpretable graph contrastive learning method with adaptive positive sampling for scRNA-seq data analysis
Source: Bioinformatics. 2025 Jul 21;41(8):btaf411. doi: 10.1093/bioinformatics/btaf411 (PMC12342183; doi:10.1093/bioinformatics/btaf411)
Supplement: btaf411_Supplementary_Data [file btaf411_supplementary_data.pdf]

Supplementary data to 'IGCLAPS: an interpretable graph contrastive learning method with adaptive positive sampling for scRNA-seq data analysis'

Weihua Zheng, Wenwen Min and Shunfang Wang

May 7, 2025

1 Availability of datasets

In this article, twelve real datasets are used for evaluation, i.e. Darmanis, LaManno, Muraro, Bladder, Adam, Zanini, Colquitt, PBMC, Young, Baron human, Baron mouse and Chen. The description and availability of these data are displayed as below:

Table S1 Description and availability of the datasets.

Name	Cells	Genes	Groups	Zero proportion	Source	Ref
Darmanis	466	22083	9	0.808	GSE67835	(Darmanis et al., 2015)
LaManno	1715	18539	17	0.804	GSE76381	(LaManno et al., 2016)
Baron human	8569	20125	14	0.906	GSE84133	(Baron et al., 2016)
Baron mouse	1886	14878	13	0.890	GSE84133	(Baron et al., 2016)
Muraro	2122	19046	9	0.730	GSE85241	(Muraro et al., 2016)
Bladder	2746	20670	16	0.949	GSE108097	(Han et al., 2018)
Adam	3660	23797	8	0.924	GSE94333	(Adam et al., 2017)
Zanini	4052	18072	15	0.822	GSE147668	(Zanini et al., 2023)
Colquitt	4110	19848	27	0.967	GSE150486	(Colquitt et al., 2021)
PBMC	4340	33694	8	0.961	10X Genomics	(Zheng et al., 2017)
Young	5685	33658	11	0.947	European Genome-phenome Archive	(Young et al., 2018)
Chen	12089	23284	46	0.937	GSE87544	(Chen et al., 2017)

2 Availability of clustering methods for comparison

In this article, nine popular clustering methods for scRNA-seq data are used for comparison in terms of clustering performance and visualization. The availability of these methods are as follows (except KMeans which is a basic method in both R and Python):

Table S2 Availability of the methods for comparison.

Methods	Source	Ref
CIDR	https://github.com/VCCRI/CIDR	(Lin et al., 2017)
ADClust	https://github.com/biomed-AI/ADClust	(Zeng et al., 2022)
scDeepCluster	https://github.com/ttgump/scDeepCluster/tree/master	(Tian et al., 2019)
scGNN	https://github.com/juexinwang/scGNN/tree/master	(Wang et al., 2021)
scMAE	https://github.com/CSUBioGroup/scMAE/tree/main	(Fang et al., 2024)
scGAC	https://github.com/Joye9285/scGAC/tree/main	(Cheng and Ma, 2022)
scCCL	https://github.com/LuckyxiaoLin/ScCCL/tree/main	(Du et al., 2023)
scDCCA	https://github.com/WJ319/scDCCA	(Wang et al., 2023)

3 Data preprocessing

IGCLAPS takes a raw expression count matrix as input. After quality control which excludes cells expressing less than 5 genes and genes expressed in less than 5 cells, we further generate the normalized expression matrix $\mathbf{Y} = (y_{ij})_{m\times n}$ with m cells and n genes, which is defined as

$$y_{ij} = \log_{10}(\frac{x_{ij}}{\sum_{k=1}^{n} x_{ik}} \times 1000000 + 1), i = 1, ..., m, k = 1, ..., n. \qquad (1)$$

Then we calculate the coefficient of variance (CV) of each normalized gene:

$$CV(Y_j) = \frac{sd(Y_j)}{\bar{Y}_j}, j = 1, ..., n, \qquad (2)$$

in which $sd(Y_j)$ and $\bar{Y}_j$ mean the standard deviation and mean value of Y_j respectively. Then, the 3000 genes with highest CV are used for KNN graph construction and further clustering.

Before constructing the KNN graph, we first perform PCA on the preprocessed expression matrix denoted by Y' and retain 50 principal components. Denote this low-dimensional matrix by Z, the cosine similarity between Z_i and Z_j can be calculated by

$$S_{ij} = \frac{Z_i \cdot Z_j}{||Z_i||||Z_j||}, i, j = 1, ..., m. \qquad (3)$$

Then the KNN graph can be expressed by its corresponding adjacency matrix A with

$$A_{ij} = \begin{cases} 1, & if\ Z_j\ is\ in\ the\ k-nearest\ neighbors\ of\ Z_i, \\ 0, & otherwise. \end{cases} \tag{4}$$

4 Evaluation metrics

In this article, adjusted Rand Index (ARI) (Hubert and Arabie, 1985), normalized mutual information (NMI) (Strehl and Ghosh, 2002) and Accuracy (ACC) are used us evaluation metrics to assess the clustering performance. ARI and NMI are formulated as:

$$ARI(A^*, A) = \frac{\sum_{i,j}\binom{N_{ij}}{2} - [\sum_i \binom{N_i}{2}\sum_j \binom{N_j}{2}]/\binom{N}{2}}{\frac{1}{2}[\sum_i \binom{N_i}{2} + \sum_j \binom{N_j}{2}] - [\sum_i \binom{N_i}{2}\sum_j \binom{N_j}{2}]/\binom{N}{2}}, \tag{5}$$

where N is the number of cells, N_{ij} is the number of cells of the real cell type $C_j^* \in A^*$ assgined to cluster C_i in partition A, and N_j is the number of cells of cell type C_j^*. The value range of ARI is $[-1, 1]$, a higher ARI indicates better clustering results, and ARI equals to 1 only when the clustering result is identical to the real cell type partition. NMI is defined as follows:

$$NMI(A^*, A) = \frac{2 \times I(A^*, A)}{H(A^*) + H(A)}, \tag{6}$$

in which $I(A^*, A) = H(A^*) - H(A^*|A)$, $H(A) = -\sum_{a \in A} p(a)log2(p(a))$, $H(A^*|A) = H(A^*, A) - H(A)$ and $H(A^*, A) = -\sum_{a^*,a} p(a^*, a)log2(p(a^*, a))$. The value of NMI falls between $[0, 1]$ and a larger NMI means better clustering performance. ACC is defined as:

$$ACC = \frac{Number\ of\ truly\ mapped\ samples}{total\ sample\ size}. \tag{7}$$

In the ablation study section, positive predicted values (PPV) and negative predicted values (NPV) are used to evaluate the performance of APS module in dividing positive and negative samples. Consider the confusion matrix of sample partition:

	True positive	True negative
Predicted positive	TP	FP
Predicted negative	FN	TN

Then PPV and NPV can be calculated as follows:

$$PPV = \frac{TP}{TP + FP}, \tag{8}$$

$$NPV = \frac{TN}{TN + FN}. \tag{9}$$

5 Supplementary tables and figures

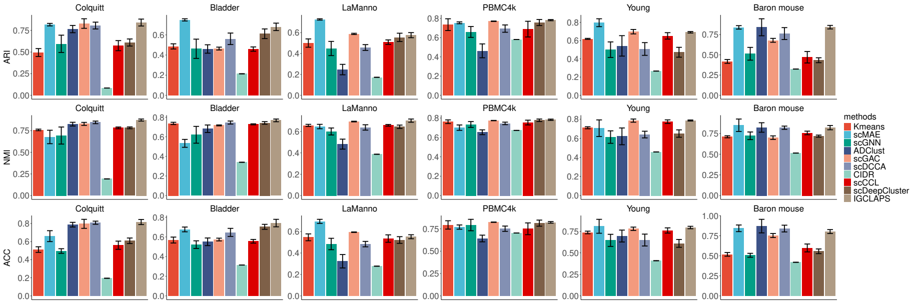

Figure S1 Clustering results of different methods measured by ARI, NMI and ACC. Higher values indicate better results.

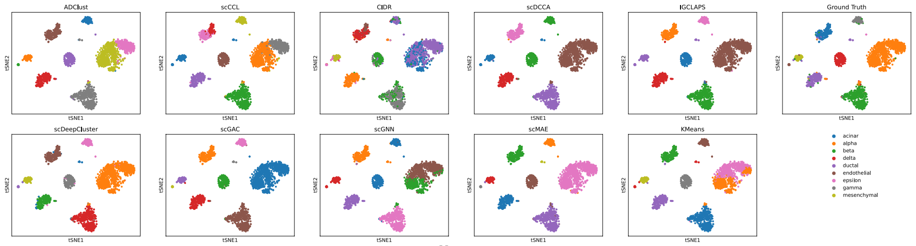

Muraro

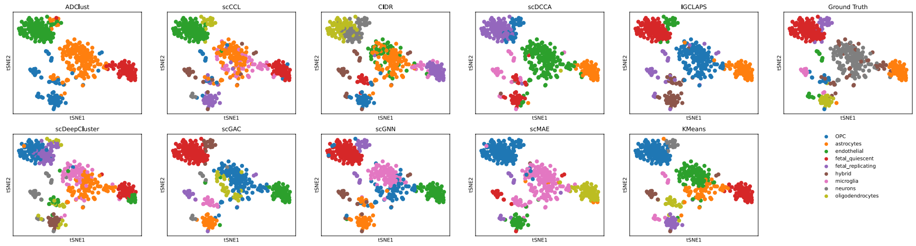

Darmanis

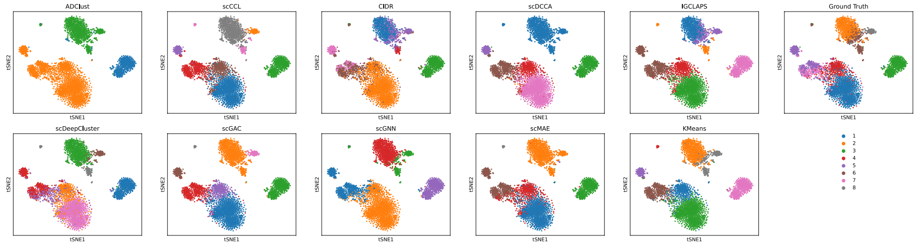

PBMC

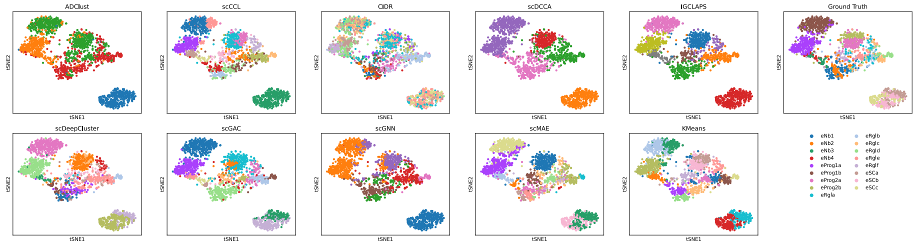

LaManno

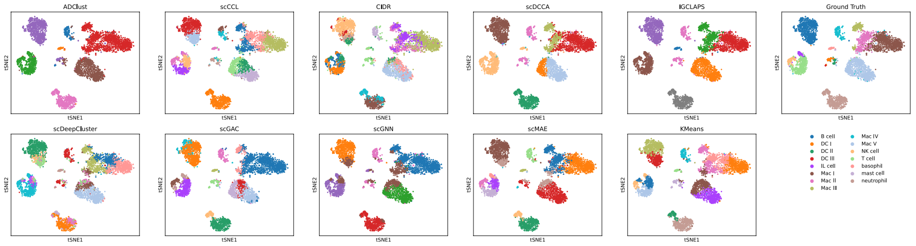

Zanini

(Continued)

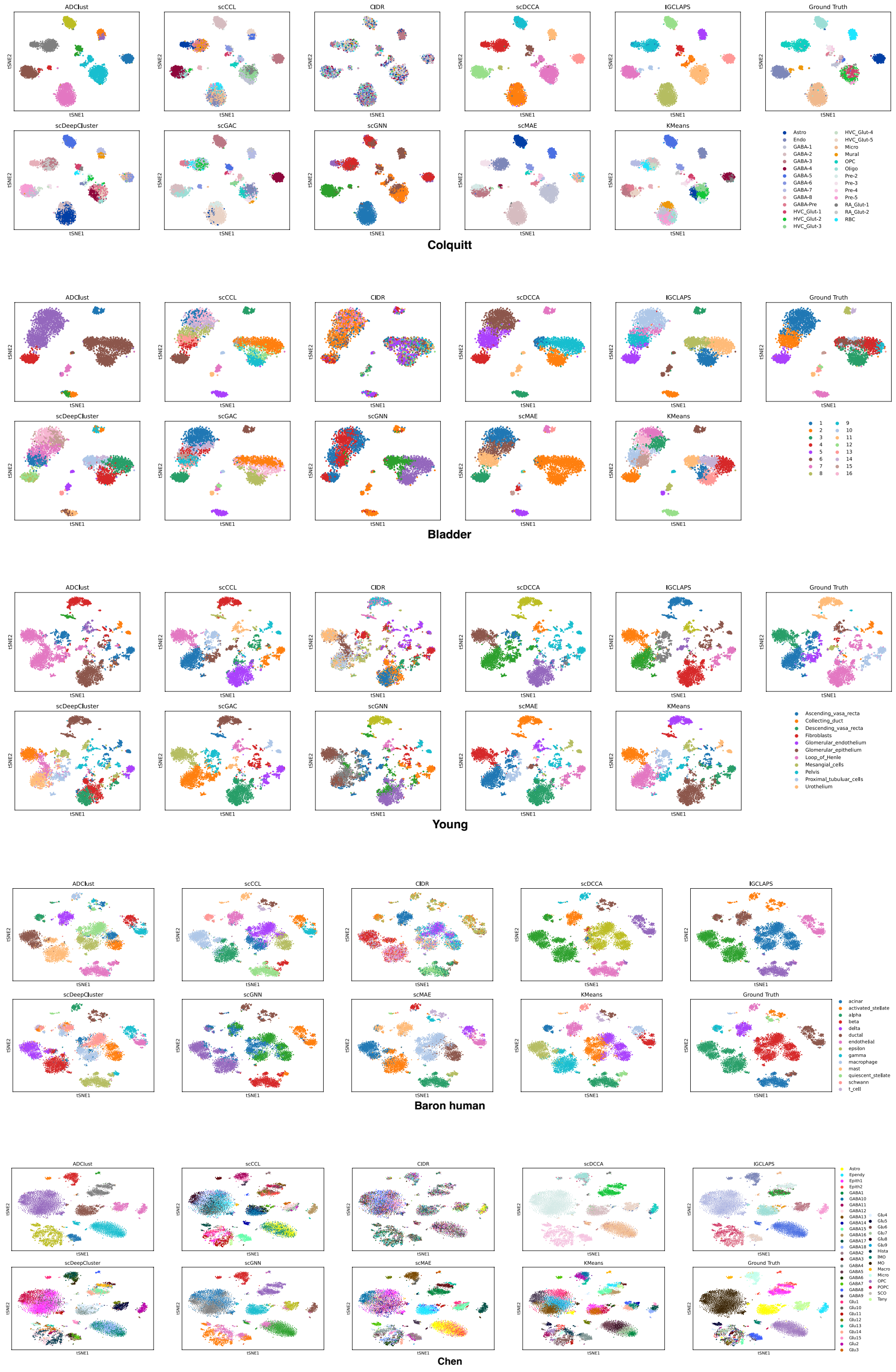

Figure S2 T-SNE visualization results of the datasets.

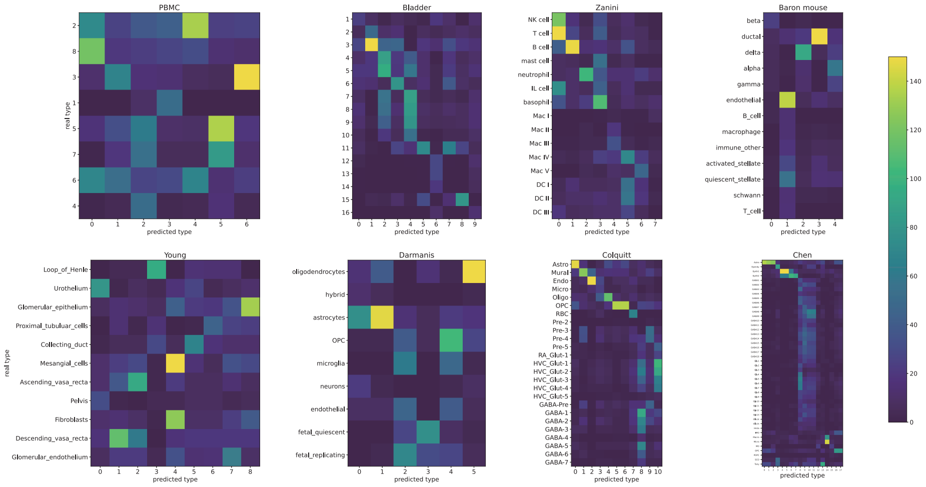

Figure S3 Heatmap of the overlapped DEGs found by IGCLAPS and Seurat.

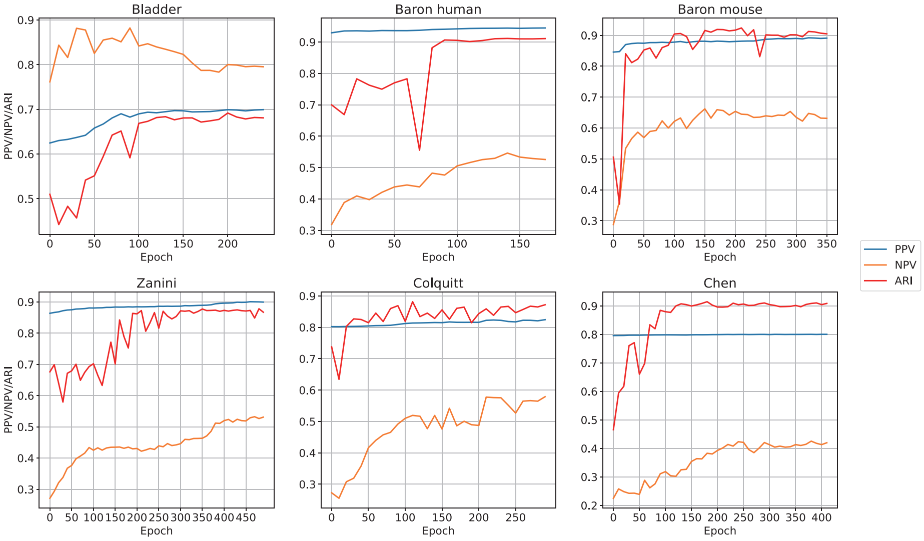

Figure S4 PPV and NPV of positive and negative samples identified by IG-CLAPS.

Table S3 Results of ablation test comparing IGCLAPS and the ablated models in terms of NMI and ACC (upper: NMI, lower: ACC). Best results are marked in bold.

neighbors	cluster-head	APS	Darmanis	LaManno	Muraro	Bladder	Adam	Zanini	Colquitt	PBMC	Young	Baron-h	Baron-m	Chen
✓	✓	✓	**0.765**	**0.702**	**0.852**	**0.768**	**0.888**	**0.869**	0.872	**0.782**	**0.786**	**0.869**	0.821	0.814
✓	✓	×	0.738	0.700	0.840	**0.768**	0.848	0.859	**0.873**	0.767	0.779	0.863	**0.838**	**0.822**
×	✓	×	0.748	0.654	0.771	0.751	0.806	0.847	0.832	0.709	0.761	0.761	0.807	0.791
✓	×	×	0.741	0.672	0.787	0.732	0.833	0.790	0.832	0.765	0.750	0.802	0.663	0.714
✓	✓	✓	**0.812**	**0.552**	**0.911**	**0.741**	**0.919**	**0.839**	**0.816**	0.822	**0.794**	**0.874**	0.802	**0.798**
✓	✓	×	0.772	0.512	0.823	0.658	0.854	0.774	0.751	0.681	0.746	0.745	0.795	0.717
×	✓	×	0.735	0.524	0.894	0.702	0.895	0.823	0.815	0.802	0.789	0.857	**0.824**	0.788
✓	×	×	0.791	0.463	0.802	0.635	0.867	0.679	0.776	**0.828**	0.756	0.739	0.629	0.508

Table S4 Clustering results with different masking proportion at the data augmentation stage.

Metrics	Mask	Darmanis	PBMC	Adam	Muraro	LaManno	Colquitt	Bladder	Young	Baron-h	Baron-m	Zanini	Chen
ARI	0.5	0.765	0.782	0.886	0.900	0.576	0.860	0.680	0.692	0.912	0.842	0.852	0.898
	0.6	0.730	0.782	0.838	0.879	0.561	0.825	0.636	0.693	0.818	0.843	0.796	0.862
	0.7	0.729	0.771	0.841	0.874	0.567	0.827	0.622	0.696	0.866	0.840	0.834	0.888
	0.8	0.684	0.742	0.872	0.896	0.558	0.775	0.500	0.687	0.910	0.827	0.856	0.798
	0.9	0.677	0.669	0.812	0.901	0.500	0.720	0.515	0.589	0.796	0.690	0.739	0.786
NMI	0.5	0.765	0.782	0.888	0.852	0.702	0.872	0.768	0.786	0.869	0.821	0.869	0.814
	0.6	0.739	0.779	0.864	0.838	0.700	0.856	0.771	0.787	0.846	0.813	0.859	0.811
	0.7	0.739	0.774	0.865	0.842	0.700	0.847	0.761	0.787	0.853	0.814	0.863	0.817
	0.8	0.710	0.767	0.879	0.849	0.693	0.834	0.698	0.774	0.866	0.788	0.875	0.797
	0.9	0.711	0.733	0.849	0.850	0.670	0.812	0.717	0.713	0.805	0.727	0.835	0.772
ACC	0.5	0.812	0.822	0.919	0.911	0.552	0.816	0.741	0.794	0.874	0.802	0.839	0.798
	0.6	0.776	0.820	0.878	0.889	0.532	0.791	0.703	0.787	0.789	0.798	0.802	0.767
	0.7	0.779	0.814	0.890	0.886	0.535	0.794	0.677	0.797	0.833	0.799	0.83	0.785
	0.8	0.732	0.801	0.914	0.900	0.531	0.772	0.584	0.790	0.874	0.782	0.854	0.716
	0.9	0.725	0.729	0.858	0.907	0.503	0.755	0.605	0.730	0.807	0.750	0.788	0.723

Table S5 Clustering results of IGCLAPS and KMeans with unknown number of cell types. ARI and NMI are used as evaluation metrics.

Metrics	Number of cell types	Darmanis	PBMC	Adam	Muraro	LaManno	Colquitt	Bladder	Young	Baron-h	Baron-m	Zanini	Chen
ARI	Known	0.765	0.782	0.886	0.900	0.576	0.860	0.680	0.692	0.912	0.842	0.852	0.898
	Unknown	0.695	0.767	0.761	0.865	0.570	0.814	0.639	0.654	0.912	0.839	0.867	0.891
	Unknown-KMeans	0.686	0.708	0.466	0.796	0.520	0.503	0.510	0.623	0.626	0.461	0.579	0.323
NMI	Known	0.765	0.782	0.888	0.852	0.702	0.872	0.768	0.786	0.869	0.821	0.869	0.812
	Unknown	0.725	0.780	0.817	0.837	0.703	0.857	0.770	0.757	0.873	0.809	0.872	0.818
	Unknown-KMeans	0.724	0.750	0.610	0.821	0.661	0.761	0.741	0.687	0.787	0.721	0.734	0.667

6 Time cost of different methods

In this section, we display the time consumption of different methods on all twelve real data used in this article. All methods are implemented on an RTX 4090D GPU for ten times and the average time costs are shown below.

Table S6 Time consumption measured in seconds of different methods for cell clustering.

Data\method	IGCLAPS	scDeepCluster	scCCL	scGAC	CIDR	scGNN	KMeans	scMAE	ADClust	scDCCA
Darmanis	43±5.3s	102±9.2s	36±3.2s	39±5.5s	2.1±0.3s	220±26.3s	≤1s	18±2.2s	27±4.0s	43±5.1s
LaManno	65±5.2s	264±41.9s	77±8.3s	53±6.0s	14±1.9s	383±44.2s	≤1s	72±5.5s	43±5.4s	152±22.0s
Bladder	83±9.3s	397±55.7s	110±8.2s	107±11.4s	47±8.8s	426±60.0s	≤1s	104±11.2s	68±3.9s	239±20.8s
Muraro	31±2.9s	264±22.4s	82±7.1s	53±6.6s	25±1.9s	294±22.4s	≤1s	34±4.0s	73±8.2s	188±20.0s
Adam	96±13.9s	580±112.5s	140±17.7s	133±19.2s	80±10.1s	365±55.3s	≤1s	131±19.8s	86±15.2s	312±28.5s
Zanini	93±10.3s	680±142.0s	149±20.3s	230±31.3s	112±16.6s	460±71.1s	≤1s	68±6.9s	139±8.2s	349±50.0s
Colquitt	165±22.4s	303±37.8s	167±15.0s	126±14.2s	75±10.0s	495±66.5s	≤1s	198±10.1s	108±9.4s	359±47.2s
PBMC	168±14.4s	457±56.3s	155±22.7s	200±28.0s	128±12.8s	429±60.4s	≤1s	91±7.3s	174±20.2s	370±80.9s
Young	152±19.8s	958±117.4s	192±20.0s	416±61.1s	274±39.0s	482±80.4s	≤1s	122±10.7s	159±11.9s	493±52.2s
Baron human	169±17.0s	1039±224.9s	283±21.5s	nan	695±88.1s	642±80.9s	≤1s	172±23.8s	338±29.9s	685±92.0s
Baron mouse	78±12.5s	144±19.2s	82±7.7s	94±11.2s	15±0.8s	289±27.4s	≤1s	30±4.2s	72±11.0s	160±21.1s
Chen	280±39.3s	1706±284.4s	511±62.3s	nan	1848±177.5s	862±129.4s	≤1s	321±42.0s	103±10.2s	1756±201.8s

References

Adam M, Potter A.S and Potter S.S. Psychrophilic proteases dramatically reduce single-cell RNA-seq artifacts: a molecular atlas of kidney development. *Development*, 144(19):3625–3632, 2017.

Baron M, Veres A, Wolock S.L et al. Single-cell transcriptomic map of the human and mouse pancreas reveals inter-and intra-cell population structure. *Cell Syst*, 3(4):346–360, 2016.

Chen R, Wu X, Jiang L et al. Single-cell RNA-seq reveals hypothalamic cell diversity. *Cell Rep*, 18(13):3227–3241, 2017.

Cheng Y and Ma X. scGAC: a graph attentional architecture for clustering single-cell RNA-seq data. *Bioinformatics*, 38(8):2187–2193, 2022.

Colquitt B.M, Merullo D.P, Konopka G et al. Cellular transcriptomics reveals evolutionary identities of songbird vocal circuits. *Science*, 371(6530):eabd9704, 2021.

Darmanis S, Sloan S.A, Zhang Y et al. A survey of human brain transcriptome diversity at the single cell level. *Proc Natl Acad Sci U S A*, 112(23): 7285–7290, 2015.

Du L, Han R, Liu B et al. ScCCL: Single-Cell Data Clustering Based on Self-Supervised Contrastive Learning. *IEEE/ACM Trans Comput Biol Bioinform*, 20(3):2233–2241, 2023.

Fang Z, Zheng R, Li M. scMAE: a masked autoencoder for single-cell RNA-seq clustering. Bioinformatics. *Bioinformatics*, 40(1):btae020. 2024.

Han X, Wang R, Zhou Y et al. Mapping the mouse cell atlas by microwell-seq. *Cell*, 127(5):1091–1107, 2018.

Hubert, L. and Arabie, P. Comparing partitions. *J. Classif*, 2(1), 193–218, 1985.

La Manno G, Gyllborg D, Codeluppi S et al. Molecular diversity of midbrain development in mouse, human, and stem cells. *Cell*, 167(2):566–580, 2016.

Lin P, Troup M and Ho J.W. CIDR: Ultrafast and accurate clustering through imputation for single-cell RNA-seq data. *Genome Biol*, 18:59, 2017.

Muraro M.J, Dharmadhikari G, de Koning E et al. A single-cell transcriptome atlas of the human pancreas. *Cell Syst*, 3(4):385–394, 2016.

Strehl, A. and Ghosh, J. Cluster ensembles - a knowledge reuse framework for combining multiple partitions. *J. Mach. Learn. Res*, 3: 583–617, 2002.

Tian T, Wan J and Song Q et al. Clustering single-cell rna-seq data with a model-based deep learning approach. *Nat Mach Intell* 1:191–198, 2019.

Wang J, Ma A, Chang Y et al. scGNN is a novel graph neural network framework for single-cell RNA-Seq analyses. *Nat Commun*, 12:1882, 2021.

Wang J, Xia J, Wang H, Su Y et al. scDCCA: deep contrastive clustering for single-cell RNA-seq data based on auto-encoder network. *Brief Bioinform*, 24(1):bbac625, 2023.

Wolf F, Angerer P and Theis F. SCANPY: large-scale single-cell gene expression data analysis. *Genome Biol*, 19:15, 2018.

Young M.D, Mitchell T.J, Vieira Braga F.A et al. Single-cell transcriptomes from human kidneys reveal the cellular identity of renal tumors. *Science*, 361(6402):594–599, 2018.

Zanini F, Che X, Knutsen C et al. Developmental diversity and unique sensitivity to injury of lung endothelial subtypes during postnatal growth. *iScience*, 26(3):106097, 2023.

Zeng Y, Wei Z, Zhong F et al. A parameter-free deep embedded clustering method for single-cell RNA-seq data. *Brief Bioinform*, 23(5):bbac172, 2022.

Zheng G, Terry J.M, Belgrader P et al. Massively parallel digital transcriptional profiling of single cells. *Nat Commun*, 8:14049, 2017.
